# Tocilizumab for Severe COVID-19 Infection and Multisystem Inflammatory Syndrome in Adults and Children

**DOI:** 10.3390/life13040889

**Published:** 2023-03-27

**Authors:** Amber N. Edinoff, Edward Sanders Alpaugh, Olivia Newgaard, Irza Wajid, Rachel J. Klapper, Elyse M. Cornett, Adam M. Kaye, Praneet Iyer, Alan D. Kaye

**Affiliations:** 1 Department of Psychiatry and Behavioral Medicine, Louisiana State University Health Sciences Center at Shreveport, Shreveport, LA 71103, USA; 2 Department of Anesthesiology, Louisiana State University Health Sciences Center at New Orleans, New Orleans, LA 70112, USA; 3 Medical College of Wisconsin, School of Medicine, Milwaukee, WI 53226, USA; 4 Department of Radiology, Louisiana State University Health Sciences Center at Shreveport, Shreveport, LA 71103, USA; 5 Department of Anesthesiology, Louisiana State University Health Sciences Center at Shreveport, Shreveport, LA 71103, USA; 6 Department of Pharmacy Practice, Thomas J. Long School of Pharmacy and Health Sciences, University of the Pacific, Stockton, CA 95211, USA; 7 Department of Pulmonary and Critical Care, Baptist Memorial Hospital, Desoto, MS 38671, USA

**Keywords:** tocilizumab, COVID-19, acute respiratory distress syndrome, IL-6

## Abstract

Coronavirus disease 2019 (COVID-19) rapidly emerged as a global pandemic, placing imminent stress and burden on healthcare resources and workers worldwide. Many patients who present with a severe COVID-19 infection are at high risk of developing severe acute respiratory distress syndrome (ARDS), leading to a vast number of patients requiring mechanical ventilation and a high mortality rate. Similar to Middle East respiratory syndrome, COVID-19 demonstrates an initial viral replication phase that manifests as a variety of symptoms typically flu-like in nature, followed by a profound inflammatory response leading to rapid production of cytokines and uncontrolled inflammation. There have also been many cases of COVID-19 in pediatric patients presenting with elevated inflammatory markers and multisystem involvement labeled as a multisystem inflammatory syndrome (MIS-C) by the world health organization (WHO). The recent treatment of systemic inflammatory response to COVID-19 targets the secondary phase involving cytokine release syndrome. The detrimental effects of IL-6 can be profound and elevated levels are associated with a higher mortality rate and mechanical ventilation. Tocilizumab is an IL-6 inhibitor most widely investigated to target cytokine storm syndrome. Since June 2021, the FDA enacted an emergency use authorization for tocilizumab in the treatment of COVID-19. Several clinical trials have investigated tocilizumab combined with corticosteroids for treating severe ARDS associated with COVID-19. An increasing amount of evidence suggests that targeting the cytokine storm syndrome related to COVID-19 can lead to improved outcomes, especially in those patients requiring mechanical ventilation and with a critical illness. Additional studies are warranted to further look at the positive effects of tocilizumab in the COVID-19 population while additionally defining possible adverse effects.

## 1. Introduction

Coronavirus disease 2019 (COVID-19) rapidly emerged as a global pandemic, placing significant stress and burden on healthcare resources and workers worldwide. Many patients who present with a severe COVID-19 infection are at high risk of developing severe acute respiratory distress syndrome (ARDS), leading to a vast number of patients requiring mechanical ventilation and a high mortality rate [1] (p. 19). It is estimated that 67% of COVID-19 patients with severe infection develop ARDS [2]. Similar to Middle East respiratory syndrome, COVID-19 demonstrates an initial viral replication phase that manifests as a variety of symptoms typically flu-like in nature, followed by a profound inflammatory response leading to the rapid production of cytokines and uncontrolled inflammation. This second inflammatory phase demonstrated an increased propensity to result in ARDS and multiple organ failure [1,3]. Children are susceptible to the virus and a new disease presentation has been labeled as a multisystem inflammatory syndrome (MIS-C in children, and MIS-A in adults) by the World Health Organization (WHO). MIS-C has emerged in children 4–6 weeks after being affected by COVID-19 that has been shown to have comparable death rates to adults with severe COVID-19 [4,5]. This syndrome is associated with various illnesses, including Kawasaki disease (KD), macrophage activation syndrome, Kawasaki shock syndrome, and toxic shock syndrome [6]. The Centers for Disease Control and Prevention (CDC) developed a working definition for MIS-A that includes age 21 years or old, the presence of a severe illness requiring hospitalization, a recent positive COVID-19 test result, severe extrapulmonary organ system dysfunction, markedly elevated acute inflammatory markers, and/or absence of severe respiratory illness, which excludes patients in whom tissue hypoxia causes organ system dysfunction [7].

While SARS-CoV-2 can lead to respiratory failure, septic shock, and ARDS, the spectrum of the illness includes asymptomatic/ presymptomatic, mild, moderate, severe, and critical cases [8]. Asymptomatic/presymptomatic infections include individuals who test positive for SARS-CoV-2 but have no symptoms consistent with COVID-19 [8]. Mild illness includes individuals who have various signs and symptoms of COVID-19 but do not have shortness of breath, dyspnea, or abnormal chest imaging [8]. Moderate illness is when a patient shows evidence of lower respiratory disease during clinical assessment or imaging and has an oxygen saturation (SpO_2_) of greater than or equal to 94% [8]. Severe illness is found in individuals who have a SpO_2_ < 94% on room air, a ratio of arterial partial pressure of oxygen to the fraction of inspired oxygen (PaO_2_/FiO_2_) of <300 mm Hg, a respiratory rate > 30 breaths/min, or lung infiltrates >50% [8]. Critical illness is found in individuals who have respiratory failure, septic shock, and/or multiple organ dysfunction [8].

The recent treatment of systemic inflammatory response to COVID-19 targets the secondary phase involving cytokine release syndrome. Evidence has shown that various acute phase reactants and markers of inflammation, including interleukin-6 (IL-6), ferritin, and C-reactive protein (CRP), are elevated. More specifically, IL-6 has been associated with a higher viral load, prolonged viral RNA shedding, as well as poorer outcomes, including respiratory failure and death [9] (p. 19). Hypercytokinemia can also explain thromboinflammation and microthrombi associated with endotheliitis in patients with COVID-19. IL-6 has been shown to potentiate the coagulation cascade by causing platelet adhesion through increased production of selectins in endothelial cells, in addition to leading to the increased production of factor VIIa [10,11].

Furthermore, IL-6 can lead to a deregulated immune response with the lymphocytic infiltration of the lung parenchyma through monocyte and neutrophil infiltration causing tissue damage [9] (p. 19). Currently, IL-6 and its antagonists have been trialed for their efficacy, showing some positive results, including the REMAP-CAP and RECOVERY trials [12]. Tocilizumab is an IL-6 inhibitor most widely investigated to target cytokine storm syndrome. Since June 2021, the FDA enacted an emergency use authorization for tocilizumab for the treatment of COVID-19. Since there is inconsistent evidence of improvement in clinical outcomes and mortality, the FDA does not have approval for the use of tocilizumab and recommends careful consideration in specific patient populations [3,11]. While tocilizumab has a low side effect profile, it may serve as a useful adjunctive treatment with supportive care and corticosteroid use for severe COVID-19 pneumonia, MIS-C, and MIS-A [13].

## 2. Multisystem Inflammatory Syndrome

### 2.1. Causes 

SARS-CoV-2 is a viral infection that enters a host protein by binding its spike protein to the human ACE2 receptor. It was originally predicted that COVID-19 would not affect children, whether that be due to lower levels of ACE2 receptors or a more robust humoral immune response. Cases of MIS-C have been reported in the United Kingdom, Italy, Spain, France, Switzerland, and the United States [14]. Although it is still unknown if the same clinical etiologies that lead to respiratory failure and shock with SARS-CoV-2 also lead to the shock associated with MIS-C, many have concluded that the most likely cause of MIS-C is a post-infectious, immunologically mediated hyperinflammatory response to COVID-19 [4,13]. SARS-CoV-2 has not been proven to be the definitive cause of MIS-C; however, the sudden appearance during the global pandemic and positive serum anti-SARS-CoV-2 antibodies in most patients is highly likely to have a high impact indicating that the two diseases are connected [14]. 

### 2.2. Pathophysiology

While the pathogenesis of MIS-C is still unknown, it is strongly suspected to be due to vasculitis and an autoimmune etiology [15]. In the Quantitative SARS-CoV-2 Serology study of MIS-C, Rostad et al. reported that children with MIS-C had significantly higher levels of SARS-CoV-2 receptor binding domain immunoglobulin G (IgG) antibody titers than those with COVID-19, KD, and hospital controls. They also found that titer levels were correlated with prolonged hospital and ICU lengths of stay [16]. Both KD and MIS-C tend to show increased levels of TNF-α. However, KD is most associated with elevations in IL-1, IL-2, and IL-6.

In contrast, the hypercytokinemia found in MIS-C patients is believed to have a very specific hyperinflammatory process that is not associated with the cytokines IL-1R, IL-2, IL-4, IL-12p70, or IL-13 [17]. Diorio et al. found that, unlike patients with severe COVID-19, patients with MIS-C were noted to have burr cells, elevated IL-10 and TNF-α, rare schistocytes, toxic granulation on peripheral blood smears, and leukocytopenia, and many had an increased number of immature myeloid cells [17]. Consiglio et al. found that IL-17A mediates hyperinflammation in KD but not in MIS-C. They also concluded that MIS-C has a much more diffuse endothelial involvement and immunopathology due to the higher levels of biomarkers associated with arthritis and coronary artery disease than KD [15]. Kabeerdoss et al. proposed the following immunological mechanism of MIS-C: “after infection with SARS-CoV-2. Those genetically susceptible may produce viral-specific antibodies that may be cross-reactive with host antigens bind to Fcγ receptors in neutrophils and macrophages. This may lead to the secretion of pro-inflammatory cytokines leading to the clinical manifestation of MIS-C” [4]. In conclusion, while the pathogenesis of MIS-C is still unknown, laboratory findings in patients with MIS-C suggest a high probability of an autoimmune etiology. 

MIS-A has been described in the literature as very similar to Kawasaki disease seen in children and shares many of the same clinical features as MIS-C. Cases have been described of adults having a Kawasaki-like illness in the context of a COVID-19 infection [15] (p. 19) [18]. These cases have been termed MIS-A, as they share similar symptomatology to MIS-C. This similar symptomatology suggests a similar pathophysiology of an autoimmune process in MIS-A.

### 2.3. Epidemiology

There is still a lot of unknown information regarding the epidemiology of MIS-C. Interestingly, it has been shown that patients who present with MIS-C were otherwise previously healthy children [13,19]. While initial studies found that less than 5% of children affected by SARS-CoV-2 experienced severe effects of the disease, the risk of the severe hyperinflammatory disease MIS-C is not to be ignored. According to the CDC, as of October 2020, there have been over 1000 confirmed MIS-C cases and 20 deaths related to MIS-C [20]. Gender is not a risk factor. However, race does seem to play a possible role in epidemiology. In a review of over 371 articles by Ahmed et al., including 662 children with MIS-C, children of African American, Afro-Caribbean, or African race/ethnicity represented 34.7% of the population [5]. MIS-C also tends to affect older children and adolescents [14]. It has also been postulated that there may be a genetic component in MIS-C development [4]. In terms of MIS-A, it is unclear who will develop this syndrome and who will not at this time.

### 2.4. Treatment

The main goal of treatment is to stabilize the patient and prevent long-term cardiac sequelae [21]. Vital signs, hydration, electrolytes, and metabolic status need to be continuously monitored [22]. Empiric therapy with broad-spectrum antibiotics has also been a first-step approach to treating MIS-C [20]. Hypotensive shock is a risk factor for MIS-C. It should be treated with epinephrine as a first-line treatment, IV fluids, other vasopressors, and possibly dobutamine due to the drug’s selective inotropic effects [22]. Data show that vasopressors are needed in about 66% of cases of MIS-C due to marked hypotension, and inotropic therapy is used in about 67% of patients with the disease [18,20].

Due to the similar presentation and diagnostic criteria of KD, most MIS-C cases are treated using the standard protocol for KD. This protocol includes treatment with acetylsalicylic acid (ASA), intravenous immunoglobin (IVIG), and corticosteroids. A multidisciplinary team consisting of pediatric rheumatologists, adult rheumatologists, pediatric cardiologists, pediatric infectious disease specialists, and pediatric critical care physicians, collectively termed “The Task Force”, has studied the treatment plans of MIS-C to date and devised a recommended treatment protocol for patients with MIS-C [21]. The Task Force states that low-dose ASA is recommended in all MIS-C patients without active bleeding [21]. By binding and inhibiting the inducible isoform, COX-2, the inflammatory mediators that lead to rheumatologic symptoms are decreased [23]. The antiplatelet properties of ASA also make it beneficial for use in MIS-C due to the increased risk of coagulopathies. ASA toxicity has not been reported in treating patients with MIS-C [20]. The Task Force states that IVIG should be given to all MIS-C patients who require hospitalization at a 2 gm/kg dose based on body weight. IVIG is a combined blood product from thousands of donors that contains extremely high levels of immunoglobins (Igs) [24]. IVIG has many anti-inflammatory effects and proposed mechanisms. It is believed that IVIG interacts with FcγRs present on monocytes and macrophages, limiting antibody-dependent cytotoxicity and inhibiting B-cell activation. Other proposed mechanisms involve the neutralization of circulating autoantibodies and induction of a block in B-cell proliferation. IVIG also interferes with the activated complement system components and forms the membrane attack complex [24]. There are numerous beneficial effects of IVIG for managing hyperinflammation. The administration of this treatment to patients with MIS-C has been shown to lead to a rapid resolution of fever and a reduction in the inflammatory-mediated response [20]. The Task Force also states that glucocorticoids should also be given as adjunctive therapy in ill-appearing patients with elevated BNP levels, unexplained tachycardia, or who present with shock and/or organ-threatening disease [21]. Corticosteroids have long been used for treating rheumatologic disorders due to their ability to downregulate the transcription factor NF-kB, leading to fewer pro-inflammatory cells such as TNF-α. Methylprednisolone and prednisolone have been used extensively and successfully in patients with MIS-C [18,20]. 

Treatment for MIS-C is still evolving. Although there is no standardized universal guideline for the administration of treatment, the regimens recommended above have been shown to have great success in treating children affected by the disease. Treatment for MIS-A in the available case studies has been mainly vasopressors, with one case study using tocilizumab [15] (p. 19).

## 3. Tocilizumab

A variety of pharmacologic agents were assessed to treat COVID-19 respiratory symptoms, including glucocorticoids, antiviral medications, and interferon-alpha [1]. Due to the hypercytokinemia involved in the second phase of SARS-CoV-2, tocilizumab (which inhibits IL-6 receptors) has gained recent attention due to early suggestions that it may lead to improved survival in mechanically ventilated patients. Tocilizumab is a humanized monoclonal antibody that binds to plasma IL-6 receptors and membrane-bound IL-6 receptors. Binding to this receptor prevents the promotion of its associated inflammatory pathways. IL-6 has a versatile role in inflammation via the activation of acute-phase protein synthesis in the liver, the stimulation of T-cell activity, the production and secretion of immunoglobulins, and increased hematopoiesis [25]. Furthermore, preventing IL-6 from aiding in lymphocytic infiltration into the pulmonary parenchyma and its associated tissue damage may improve respiratory and multiorgan effects [9]. By inhibiting the IL-6-mediated cascade, tocilizumab may diminish the effects of the cytokine-associated syndrome in critically ill patients. 

Currently, tocilizumab is approved by the FDA for treating rheumatoid arthritis (RA), giant cell arteritis, system sclerosis-associated interstitial lung disease, polyarticular and systemic juvenile idiopathic arthritis, and cytokine release syndrome. Tocilizumab injections and infusions are safe and effective in reducing RA-associated joint damage and decreasing the overall effects of the predominant inflammatory cascade. There is also evidence that tocilizumab may normalize the QTc interval by dampening systemic inflammation, thus reducing the risk of arrhythmia in patients with RA [26]. Furthermore, while studied in patients treated for RA, tocilizumab decreased C-reactive protein to the normal range following two weeks of therapy [25]. Studies on efficacy in RA showed fewer serious adverse events than placebo, including pneumonia and infectious arthritis, although causal relationships could not be ruled out. As an inhibitor of the overall inflammatory response and component of the primary immune system, the risk of the diminished immune response could lead to a potentially life-threatening infection [27]. Infusion reactions have also been documented, including pruritus, headaches, flush, rash, arthralgia, and elevated blood pressure. These studies also revealed laboratory abnormalities, including elevated total cholesterol, triglycerides, and low-density lipoprotein cholesterol. A smaller portion of patients experienced a minor increase in baseline AST, ALT, and total bilirubin, which became stable after week 16. However, one patient in this group had to drop out of the study related to abnormal liver function [28]. While tocilizumab has associated laboratory and infusion-related side effects, it has a safe profile and already has FDA approval for multiple common inflammatory conditions associated with IL-6. 

The administration of IL-6 inhibitors should be avoided in immunosuppressed patients who, are prescribed other biologic immunomodulating drugs, have ALT greater than five times the normal limit, absolute neutrophil count (ANC) <500, platelet count <50 G/L, have a high risk for gastrointestinal perforation, and an uncontrolled infectious illness other than SARS-CoV-2 viral infection [12]. Additional treatment with a course of dexamethasone or equivalent is further recommended. One patient with a SARS-CoV-2-induced cytokine release syndrome was successfully treated with a combination of tocilizumab and remdesivir [29]. Initial studies showed conflicting results and no significant difference in mortality or clinical status between tocilizumab-treated and placebo groups. Some of these studies were restricted due to low power, inconsistency with the administration of corticosteroids, and variability in study populations [3]. 

### Pharmacokinetics/Pharmacodynamics

As previously mentioned, tocilizumab binds to both soluble and membrane-bound IL-6 receptors, causing an overall reduced inflammatory cascade of IL-6-mediated signaling. Doses for therapy in RA showed the greatest improvement with 8 mg per kg and had overall reduced rheumatoid factor, erythrocyte sedimentation rate, and serum amyloid A. Another study with healthy subjects administered doses of 2 to 28 mg/kg IV and 81 to 162 mg subcutaneously (SC). In these patients, absolute neutrophil counts dropped three to five days after administration. Additionally, the maximum serum concentration and area under the curve have been studied with this dosing. Single doses of 81 mg and 162 mg led to a 6.4-fold increase in area under the serum time curve and a fourfold increase in maximum serum concentration [30].

Drug–drug interactions can occur with the administration of tocilizumab. In vitro analysis has indicated that tocilizumab administration can reverse the IL-6 suppression of several CYP450 isoenzyme mRNA expressions. This effect can lead to increased CYP450 activity and increased metabolism of CYP450 substrates. For this reason, current recommendations suggest the close monitoring of drug levels for patients prescribed medications such as warfarin, cyclosporine, theophylline, or oral contraceptives [25]. While there are various dynamic changes to the patient’s metabolic and immunologic state, tocilizumab has been studied with different routes of administration, demonstrating safe, effective dosing for the majority of patient populations. Importantly, in patients infected with SARS-CoV-2, IL-6 blockade with tocilizumab does not impair the viral-specific antibody responses. There was a delayed viral clearance observed; however, this was likely driven by a higher initial viral load. Overall, this study supports the safety of IL-6 blockade therapy in patients with COVID-19 [31].

## 4. Clinical Studies

Niño-Taravilla et al. reported an 8-year-old who had pediatric inflammatory multisystem syndrome temporally associated with SARS-CoV-2 (PIMS-TS) [32]. PIMS-TS is a rare disease that can be difficult to diagnose, and supportive care is important for a successful outcome. The patient’s symptoms included multiorgan dysfunction, which consisted of mixed shock (distributive and cardiogenic), cardiac dysfunction with myocarditis, pneumonia, acute kidney failure, gastrointestinal inflammation, and pancreatitis. The patient was admitted to the pediatric intensive care unit (PICU). The patient was treated with tocilizumab (8 mg/kg) and corticosteroid therapy. The symptoms started to improve after 2 days with the normalization of the patient’s cardiac function and laboratory results. Vasoactive support was discontinued the day after the administration of tocilizumab, and the patient was then extubated a day later. The patient had a normal abdominal ultrasound and increased pancreatic enzymes on day 4. Prednisone was administered until ferritin levels were under 500 mg/dL, and enoxaparin prophylaxis was maintained for ten days. Antibiotic treatment was discontinued after five days. Serial echocardiograms specifically were performed to rule out coronary aneurysms and normal ventricular function. After 17 days, the patient was discharged. The patient followed up a month after discharge and remained asymptomatic. 

Celikel et al. investigated the effects of intravenous immunoglobin, corticosteroids, anakinra, and/or tocilizumab on children who were being treated in the PICU [33]. The study looked at 33 patients with MIS-C who had a positive or recent SARS-CoV-2 infection or had been exposed to COVID-19 within four weeks before the start of symptoms. The patients had a median age of 9 years. Clinical features consisted of Kawasaki disease/shock syndrome (63.6%), secondary hemophagocytic lymphohistiocytosis/macrophage activation syndrome (36.4%), myocardial dysfunction, and/or coronary artery abnormalities (54.5%). All the patients were given intravenous immunoglobin and corticosteroids. Anakinra, the interleukin-1 receptor antagonist, was given to 23 (69.6%) patients [33]. Two patients were switched to tocilizumab due to the limited response from anakinra. The addition of the biological therapy caused a significant increase in lymphocyte and platelet counts and a significant decrease in ferritin, B-type natriuretic peptide, and troponin levels at the end of the first week of treatment. The mortality rate of MIS-C patients in the PICU was 6% during the study period. 

Riollano-Cruz et al. conducted a chart review study of pediatric patients admitted to Mount Sinai Hospital from 24 April to June 2020, diagnosed with MIS-C with symptoms similar to Kawasaki disease associated with SARS-CoV-2 [34]. The review looked at 15 patients between the ages of 3 and 20 years, 11 of whom had no underlying medical conditions. All the patients tested positive for the SARS-CoV-2 antibody. Patients who showed symptoms of unstable blood pressure or clinical decompensation were given tocilizumab and other clinically recommended treatments. The study concluded that heightened IL-6 and IL-8 may be the driving force for MIS-C compared with Kawasaki disease, for which the driving force is increased in IL-1 [34]. Fourteen patients in the study showed improvement in their symptoms, and only one patient died. The importance of controlling cytokine levels and administering treatment that can control these levels is beneficial in treating critically ill children with COVID-19. 

Kaushik et al. conducted a clinical characteristic and outcome study of pediatric patients with MIS-C associated with SARS-CoV-2 among different care centers [35]. These patients were admitted into PICU between 23 April and 23 May 2020. The study observed 33 children with fever and vomiting as the common symptoms among the different demographics. A varying degree in the severity of the illness was also seen in this population. Eighteen patients received intravenous immunoglobulin, seventeen received corticosteroids, twelve received tocilizumab, seven received remdesivir under compassionate use, four received anakinra, and one received convalescent plasma therapy. The 12 patients that received tocilizumab were given this medication based on their cytokine panel, which showed heightened IL-6 levels. Most patients showed improvements on day 4 or 5 and were discharged by day 7. Inflammatory markers and myocardial function improved by days 4 to 7. Out of the 33 children observed in this study, 32 were discharged from the PICU after about 4.7 days and were in the hospital for around 7.8 days. Only one patient died after the withdrawal of care secondary to hemorrhagic stroke while on extracorporeal membrane oxygenation (ECMO) support.

Othenin-Girard et al. presented a case report in which an adult patient was admitted into the intensive care unit for the multisystem inflammatory syndrome (MIS) with symptoms of Kawasaki disease associated with SARS-CoV-2 and myocarditis with refractory cardiogenic shock [36]. On admission to the hospital, the patient’s blood work was positive for a heightened C-reactive protein level at 275 mg/L, a white blood cell count of 18.2 G/L, and fibrinogen levels of 8.5 g/L. The patient was administered 8 mg/kg of tocilizumab when his condition worsened due to developing severe myocarditis and required intubation. The patient was given intravenous immunoglobulin therapy (IVIG) of 2 g/kg/24 hours and a second dose of tocilizumab of 8 mg/kg. Initially, the patient’s symptoms improved, and the patient was extubated. However, five days later, the patient developed mononeuritis multiplex [36]. The patient required immunosuppressive therapy and, on day 45, was released from the hospital after the resolution of the neurological complication. This case study highlighted that tocilizumab, when used as an adjunctive therapy early on in patients with severe SARS-CoV-2 infection, may be beneficial. Still, there could be potential neurological complications such as mononeuritis multiplex. 

Another case involved an adult seen in the emergency department at NYU Langone Health in New York City, NY, who presented with a Kawasaki-like multisystem inflammatory syndrome with a positive SARS-CoV-2 infection [15] (p. 19). The patient was a 45-year-old male without any past medical history who presented with a positive SARS-CoV-2 test and six days of fever, sore throat, diarrhea, bilateral lower extremity pain, conjunctivitis, and diffuse exanthem. His chest X-ray showed diffuse interstitial haziness typical for pneumonia caused by COVID-19. His fever remained elevated throughout his admission. Other vital signs abnormalities included hypotension, tachycardia with episodes of atrial fibrillation with a rapid ventricular response, and minimal oxygen requirements (1–2 L/min by nasal cannula) [15] (p. 19). Electrocardiogram demonstrated ST elevates in the anterolateral leads, which led to left heart catheterization, showing no blockage. A transthoracic echocardiogram displayed the global hypokinesis of the left ventricular wall with a mild-to-moderately reduced ejection fraction of 40%. He met the American Heart Association’s criteria for Kawasaki-like multisystem inflammatory syndrome. He was treated with therapeutic doses of low molecular weight heparin, IVIG over two days, and a single IV dose of tocilizumab 400 mg [15] (p. 19). He did not require vasopressors, nor did he require an ICU level of care during his hospitalization. After tocilizumab administration, the authors noted that he showed clinical improvement as evidenced by defervescence, the resolution of tachycardia and tachypnea, improvement in his rash and conjunctivitis, and the down-trending of inflammatory markers. He was discharged nine days after his hospital admission. The authors did note that it was unclear if the patient’s improvement was due to the IV immunoglobulins or the addition of the tocilizumab.

Another case report detailed a case of a young adult woman who presented with the symptoms of Kawasaki disease associated with severe myocarditis, ARDS, and hemodynamic instability, which developed a few weeks after having transient anosmia [37]. Serologic examination showed the detection of SARS-CoV-2 antibodies, but the virus was not detected on PCR screening. This suggested a delayed immune response to a recent COVID-19 infection. She was treated with a combination of colchicine, tocilizumab, high-dose IVIG, and methylprednisolone. She obtained a full recovery after discharge from the hospital 17 days after treatment was initiated. The authors noted that although this patient had a positive response to tocilizumab regarding her ARDS and signs of cardiac dysfunction, she developed a cardiac aneurism that was detected three days later. This development of a cardiac aneurysm has been noted in other cases of KD treated with tocilizumab and notes that the detection of these developments is important. The patient was treated with steroids, and her aneurysm was resolved [37]. Table 1 provides a summary of the cases discussed in this section.

## 5. Conclusions

As the rapid spread of COVID-19 strained the medical community, many different therapeutic approaches were studied to diminish the hypercytokinemic response leading to ARDS, respiratory failure, and septic shock. IL-6 inhibitors have been FDA-approved for multiple inflammatory diseases and have shown efficacy, specifically with RA, and showed reduced inflammatory markers in lab values in studied patients [28]. For this reason, IL-6 inhibitors, more specifically tocilizumab, can be administered to patients as a treatment for the severe inflammatory response to SARS-CoV-2. Through the inhibition of IL-6-mediated pathways, patients may experience reduced respiratory symptoms via decreased lymphocytic lung parenchymal infiltration and associated tissue damage. The detrimental effects of IL-6 can be profound, and elevated levels are associated with a higher mortality rate and mechanical ventilation [9] (p. 19). 

Several clinical trials have investigated tocilizumab combined with corticosteroids in treating severe SARS-CoV-2. An increasing amount of evidence suggests that targeting the cytokine storm syndrome related to COVID-19 can lead to improved outcomes, especially those patients requiring mechanical ventilation and critical illness [13]. While some mild laboratory abnormalities and infusion-related reactions may occur, subcutaneous (SC) and intravenous (IV) administration has shown to be well tolerated. Caution should be taken in specific patient groups, especially immunosuppressed or taking other medication metabolized through the CYP450 pathway [10,23]. Authors of several studies have also noted that care should be used with patients with KD-like syndromes, including those with MIS-C and MIS-A. They could develop cardiac aneurysms with tocilizumab therapy, and close monitoring for this development is encouraged in the literature to avoid further complications.

While there is no FDA approval for tocilizumab, some studies have been shown to reduce overall mortality and requirements for mechanical ventilation in patients with severe COVID-19 pneumonia. Tocilizumab has historically been a safe medication while treating various inflammatory disorders and may be a beneficial therapy for SARS-CoV-2 and MIS in both pediatric and adult populations [33]. More studies will need to be performed to further look at the positive effects of tocilizumab in the COVID-19 population while further defining the possible adverse effects.

## Figures and Tables

**Table 1:** Clinical summaries with tocilizumab.

Author	Type	Date	Tocilizumab Dose	Number of Patients	Patient Age	Recovered	Summary
Niño-Taravilla et al.	Case report	25 November 2020	8 mg/kg	1 case	8 years old	Yes	The trial treated a PIMS-TS child. Tocilizumab and corticosteroids improved the child after two days. The study found that tocilizumab helps PIMS-TS patients reduce inflammation.
Celikel et al.	Case study	9 April 2021	8 mg/kg	33 (21 male)	2–17 years (median age 9)	2 deaths: secondary hemophagocytic lymphohistiocytosis (SHLH)/macrophage activation syndrome (MAS) 0 deaths: atypical Kawasaki disease (KD)/Kawasaki disease shock syndrome (KDSS) together with cardiac inflammation.	Biological agents were examined for SARS-CoV-2 MIS-C therapy. Treatment included intravenous immunoglobin, corticosteroids, anakinra, and tocilizumab. Patients improved in the first week of therapy. Two patients moved from anakinra tocilizumab due to poor response.
Riollano-Cruz et al.	Chart review	24 June 2021	Not provided	15 cases (11 male 4 female)	3–20 years old (mean age 12 years)	One patient expired on day 9 after admission and one remains admitted.	This study examined 15 juvenile patients admitted with SARS-CoV-2-positive multisystem inflammatory illness. Multisystem inflammatory syndrome increased IL-6 and IL-8. Tocilizumab helped 14 of 15 patients.
Kaushik et al.	Case study Systematic review	September 2020	Not provided	8 studies, 59% male	median age 7.3–10 years (	Information not provided	The study followed 33 children with MIS-C and their treatment approach. In the study, intravenous immunoglobulin, corticosteroids, tocilizumab, remdesivir, anakinra, and convalescent plasma therapy were employed. After 7.8 days, most patients were released.
Othenin-Girard et al.	Case report	11 November 2020	8 mg/kg	1 case	22-year-old male	Yes	A SARS-CoV-2-positive adult with deteriorating myocarditis and refractory cardiogenic shock was intubated and brought to the intensive care unit. IVIG and tocilizumab were used to treat MIS with Kawasaki disease. The trial shows the benefits of early adjunctive tocilizumab therapy.
Shaigany et al.	Case report	July 2020	400 mg, IV	1 case	45 years, male	Yes	A 45-year-old man with no medical history had a positive SARS-CoV-2 test and six days of fever, sore throat, diarrhea, bilateral lower extremity pain, conjunctivitis, and widespread exanthem. IVIG and a single dose of tocilizumab were prescribed for Kawasaki-like multisystem inflammatory syndrome (MIS-A). In this trial, IVIG, tocilizumab, or both improved the patient, who no longer needed ICU care and had no symptoms.
Cogan et al.	Case report	July 2020	480 mg, IV	1 case	19.9-year-old, female	Yes	After weeks of transitory anosmia, a young woman was admitted with delayed-onset KD-like disease, severe myocarditis, ARDS, and hemodynamic instability. High-dose IVIG, colchicine, and tocilizumab improved her condition. Tocilizumab treatment may have caused her heart aneurysm on day three. Steroids helped.

## Data Availability

All data and materials found in this manuscript can be found in articles found on PubMed.

## References

[1] Shi Y., Wang G., Cai X.-P., Deng J.-W., Zheng L., Zhu H.-H., Zheng M., Yang B., Yang Z. (2020). An overview of COVID-19. J. Zhejiang Univ. Sci. B.

[2] Risk Factors Analysis of COVID-19 Patients with ARDS and Prediction Based on Machine Learning|Scientific Reports. https://www.nature.com/articles/s41598-021-82492-x.

[3] Tocilizumab in Hospitalized Patients with Severe COVID-19 Pneumonia|NEJM. https://www.nejm.org/doi/full/10.1056/NEJMoa2028700.

[4] Kabeerdoss J., Pilania R.K., Karkhele R., Kumar T.S., Danda D., Singh S. (2020). Severe COVID-19, multisystem inflammatory syndrome in children, and Kawasaki disease: Immunological mechanisms, clinical manifestations and management. Rheumatol. Int..

[5] Ahmed M., Advani S., Moreira A., Zoretic S., Martinez J., Chorath K., Acosta S., Naqvi R., Burmeister-Morton F., Burmeister F. (2020). Multisystem inflammatory syndrome in children: A systematic review. EClinicalMedicine.

[6] Gupta S., Kaushik A., Sood M. (2020). Recent developments in COVID-19 therapeutics & current evidence for COVID-19-associated multisystem inflammatory syndrome. Indian J. Med. Res..

[7] Multisystem Inflammatory Syndrome in Adults (MIS-A). https://www.idsociety.org/covid-19-real-time-learning-network/disease-manifestations--complications/multisystem-inflammatory-syndrome-in-adults-mis-a/.

[8] “Clinical Spectrum,” COVID-19 Treatment Guidelines. https://www.covid19treatmentguidelines.nih.gov/overview/clinical-spectrum/.

[9] Efficacy of Tocilizumab in Patients Hospitalized with COVID-19|NEJM. https://www.nejm.org/doi/full/10.1056/nejmoa2028836.

[10] Kang S., Kishimoto T. (2021). Interplay between interleukin-6 signaling and the vascular endothelium in cytokine storms. Exp. Mol. Med..

[11] Martinelli N., Rigoni A.M., De Marchi S., Osti N., Donini M., Montagnana M., Castagna A., Pattini P., Udali S., De Franceschi L. (2022). High Plasma Levels of Activated Factor VII-Antithrombin Complex Point to Increased Tissue Factor Expression in Patients with SARS-CoV-2 Pneumonia: A Potential Link with COVID-19 Prothrombotic Diathesis. Diagnostics.

[12] “Information on COVID-19 Treatment, Prevention and Research,” COVID-19 Treatment Guidelines. https://www.covid19treatmentguidelines.nih.gov/.

[13] Tocilizumab Therapy for COVID-19: A Comparison of Subcutaneous and Intravenous Therapies—ScienceDirect. https://www.sciencedirect.com/science/article/pii/S1201971220321640.

[14] Rowley A.H. (2020). Understanding SARS-CoV-2-related multisystem inflammatory syndrome in children. Nat. Rev. Immunol..

[15] Consiglio C.R., Cotugno N., Sardh F., Pou C., Amodio D., Rodriguez L., Tan Z., Zicari S., Ruggiero A., Pascucci G.R. (2020). The Immunology of Multisystem Inflammatory Syndrome in Children with COVID-19. Cell.

[16] Rostad C.A., Chahroudi A., Mantus G., Lapp S.A., Teheran M., Macoy L., Tarquinio K.M., Basu R.K., Kao C., Linam W.M. (2020). Quantitative SARS-CoV-2 Serology in Children with Multisystem Inflammatory Syndrome (MIS-C). Pediatrics.

[17] Diorio C., Henrickson S.E., Vella L.A., McNerney K.O., Chase J., Burudpakdee C., Lee J.H., Jasen C., Balamuth F., Barrett D.M. (2020). Multisystem inflammatory syndrome in children and COVID-19 are distinct presentations of SARS–CoV-2. J. Clin. Investig..

[18] Sokolovsky S., Soni P., Hoffman T., Kahn P., Scheers-Masters J. (2021). COVID-19 associated Kawasaki-like multisystem inflammatory disease in an adult. Am. J. Emerg. Med..

[19] Shaigany S., Gnirke M., Guttmann A., Chong H., Meehan S., Raabe V., Louie E., Solitar B., Femia A. (2020). An adult with Kawasaki-like multisystem inflammatory syndrome associated with COVID-19. Lancet Lond. Engl..

[20] McMurray J.C., May J.W., Cunningham M.W., Jones O.Y. (2020). Multisystem Inflammatory Syndrome in Children (MIS-C), a Post-viral Myocarditis and Systemic Vasculitis—A Critical Review of Its Pathogenesis and Treatmen. Front. Pediatr..

[21] Henderson L.A., Canna S.W., Friedman K.G., Gorelik M., Lapidus S.K., Bassiri H., Behrens E.M., Ferris A., Kernan K.F., Schulert G.S. (2021). American College of Rheumatology Clinical Guidance for Multisystem Inflammatory Syndrome in Children Associated with SARS–CoV-2 and Hyperinflammation in Pediatric COVID-19: Version 2. Arthritis Rheumatol..

[22] Jiang L., Tang K., Levin M., Irfan O., Morris S.K., Wilson K., Klein J.D., Bhutta Z.A. (2020). COVID-19 and multisystem inflammatory syndrome in children and adolescents. Lancet Infect. Dis..

[23] Vane J.R., Botting R.M. (2003). The mechanism of action of aspirin. Thromb. Res..

[24] Perricone C., Triggianese P., Bursi R., Cafaro G., Bartoloni E., Chimenti M.S., Gerli R., Perricone R. (2021). Intravenous Immunoglobulins at the Crossroad of Autoimmunity and Viral Infections. Microorganisms.

[25] Federal Drug Administration “ACTEMRA (Tocilizumab) Injection,” FDA Prescribing Information for ACTEMRA (Tocilizumab) Injection. https://www.accessdata.fda.gov/drugsatfda_docs/label/2017/125276s114lbl.pdf.

[26] Lazzerini P.E., Acampa M., Capecchi P.L., Fineschi I., Selvi E., Moscsdelli V., Zimbone S., Gentile D., Galeazzi M., Laghi-Pasini F. (2015). Antiarrhythmic potential of anticytokine therapy in rheumatoid arthritis: Tocilizumab reduces corrected QT interval by controlling systemic inflammation. Arthritis Care Res..

[27] Tocilizumab Injection: MedlinePlus Drug Information. https://medlineplus.gov/druginfo/meds/a611004.html.

[28] Nishimoto N., Miyasaka N., Yamamoto K., Kawai S., Takeuchi T., Azuma J., Kishimoto T. (2009). Study of active controlled tocilizumab monotherapy for rheumatoid arthritis patients with an inadequate response to methotrexate (SATORI): Significant reduction in disease activity and serum vascular endothelial growth factor by IL-6 receptor inhibition therapy. Mod. Rheumatol..

[29] Ali S., Khalid S., Afridi M., Akhtar S., Khader Y.S., Akhtar H. (2021). Notes from the Field: The Combined Effects of Tocilizumab and Remdesivir in a Patient with Severe COVID-19 and Cytokine Release Syndrome. JMIR Public Health Surveill..

[30] Zhang X., Georgy A., Rowell L. (2013). Pharmacokinetics and pharmacodynamics of tocilizumab, a humanized anti-interleukin-6 receptor monoclonal antibody, following single-dose administration by subcutaneous and intravenous routes to healthy subjects. Int. J. Clin. Pharmacol. Ther..

[31] Masiá M., Fernández-González M., Padilla S., Ortega P., García J.A., Agulló V., García-Abellán J., Telenti G., Guillén L., Gutiérrez F. (2020). Impact of interleukin-6 blockade with tocilizumab on SARS-CoV-2 viral kinetics and antibody responses in patients with COVID-19: A prospective cohort study. EBioMedicine.

[32] Niño-Taravilla C., Espinosa-Vielma Y.P., Otaola-Arca H., Poli-Harlowe C., Tapia L.I., Ortiz-Fritz P. (2020). Pediatric Inflammatory Multisystem Syndrome Temporally Associated with SARS-CoV-2 Treated with Tocilizumab. Pediatr. Rep..

[33] Çelikel E., Zahide Ekici T., Fatma A., Serhat E., Emel U., Serhan Ö., Oktay P., Müge S., Nilüfer T., Serkan C. (2021). Role of Biological Agents in the Treatment of SARS-CoV-2–Associated Multisystem Inflammatory Syndrome in Children. JCR J. Clin. Rheumatol..

[34] Riollano-Cruz M., Akkoyun E., Briceno-Brito E., Kowalsky S., Reed J., Posada R., Sordillo E.M., Tosi M., Trachtman R., Paniz-Mondolfi A. (2021). Multisystem inflammatory syndrome in children related to COVID-19: A New York City experience. J. Med. Virol..

[35] Kaushik S., Aydin S.I., Derespina K.R., Bansal P.B., Kowalsky S., Trachtman R., Gillen J.K., Perez M.M., Soshnick S.H., Conway E.E. (2020). Multisystem Inflammatory Syndrome in Children Associated with Severe Acute Respiratory Syndrome Coronavirus 2 Infection (MIS-C): A Multi-institutional Study from New York City. J. Pediatr..

[36] Othenin-Girard A., Regamey J., Lamoth F., Horisberger A., Glampedakis E., Epiney J.-B., Kuntzer T., de Leval L., Carballares M., Hurni C.-A. (2020). Multisystem inflammatory syndrome with refractory cardiogenic shock due to acute myocarditis and mononeuritis multiplex after SARS-CoV-2 infection in an adult. Swiss Med. Wkly..

[37] Cogan E., Foulon P., Cappeliez O., Dolle N., Vanfraechem G., De Backer D. (2020). Multisystem Inflammatory Syndrome with Complete Kawasaki Disease Features Associated with SARS-CoV-2 Infection in a Young Adult. A Case Report. Front. Med..

